# Older Adults’ Close Friendships and Feelings of Lacking Companionship and Isolation

**DOI:** 10.1093/geroni/igaf122.1941

**Published:** 2025-12-31

**Authors:** Sarah Patterson, Yee To (Crystal) Ng, Emily Lerner, Erica Solway, Matthias Kirch, Sydney Strunk, Jeffrey Kullgren, J Scott Roberts

**Affiliations:** University of Michigan, Ann Arbor, Michigan, United States; University of Michigan, Ann Arbor, Michigan, United States; University of Michigan, Ann Arbor, Michigan, United States; University of Michigan, Ann Arbor, Michigan, United States; University of Michigan, Ann Arbor, Michigan, United States; University of Michigan, Ann Arbor, Michigan, United States; University of Michigan, Ann Arbor, Michigan, United States; University of Michigan School of Public Health, Ann Arbor, Michigan, United States

## Abstract

Having close friendships (henceforth, “friends”) in later life and their characteristics (e.g., contact, support) may impact an older adult’s feelings of companionship and isolation. We used data from the 2024 National Poll on Healthy Aging with adults ages 50 and older (N = 3,181). Logistic regression analyses indicated that the perception of not having enough friends mediates the relationship between having any friends and experiencing feelings of companionship or isolation, even after adjusting for covariates. In fully adjusted models among older adults with friends (N = 2,877), having fewer friends, less than weekly contact, and feeling that you do not have enough friends or the right amount of contact with them were each associated with being more likely to feel a lack of companionship or to feel isolated. These findings highlight the importance of satisfaction with friendships and their quality, in addition to the number of friends one has in later life.

